# Engagement With Mental Health Services Among Survivors of Firearm Injury

**DOI:** 10.1001/jamanetworkopen.2023.40246

**Published:** 2023-10-30

**Authors:** Lauren A. Magee, Damaris Ortiz, Zachary W. Adams, Brigid R. Marriott, Anthony W. Beverly, Beatrice Beverly, Matthew C. Aalsma, Sarah E. Wiehe, Megan L. Ranney

**Affiliations:** 1 Paul H. O’Neill School of Public and Environmental Affairs, Indiana University Indianapolis; 2 Department of Surgery, Indiana University School of Medicine, Indianapolis; 3 Sidney and Lois Eskenazi Hospital Smith Level One Trauma Center, Indianapolis, Indiana; 4 Adolescent Behavior Health Research Program, Indiana University School of Medicine, Indianapolis; 5 Stop the Violence Indianapolis, Indiana; 6 Children’s Health Services Research, Department of Pediatric, Indiana University School of Medicine, Indianapolis; 7 Yale School of Public Health, Yale University, New Haven, Connecticut

## Abstract

**Importance:**

Despite the prevalence of posttraumatic stress symptoms after firearm injury, little is known about how firearm injury survivors connect with mental health services.

**Objective:**

To determine facilitators and barriers to mental health care engagement among firearm injury survivors.

**Design, Setting, and Participants:**

A qualitative study of 1-on-1, semistructured interviews conducted within a community setting in Indianapolis, Indiana, between June 2021 and January 2022. Participants were recruited via community partners and snowball sampling. Participants who survived an intentional firearm injury, were shot within Indianapolis, were aged 13 years or older, and were English speaking were eligible. Participants were asked to discuss their lives after firearm injury, the emotional consequences of their injury, and their utilization patterns of mental health services. Data were analyzed from August 2022 to June 2023.

**Main Outcomes and Measures:**

Survivors’ lived experience after firearm injury, sources of emotional support, mental health utilization, and their desired engagement with mental health care after firearm injury.

**Results:**

A total of 18 participants (17 were Black [94%], 16 were male [89%], and 14 were aged between 13 and 24 years [77%]) who survived a firearm injury were interviewed. Survivors described family members, friends, and informal networks as their main source of emotional support. Barriers to mental health care utilization were perceived as a lack of benefit to services, distrust in practitioners, and fear of stigma. Credible messengers served as facilitators to mental health care. Survivors also described the emotional impact their shooting had on their families, particularly mothers, partners, and children.

**Conclusions and Relevance:**

In this study of survivors of firearm injury, findings illustrated the consequences of stigma and fear when seeking mental health care, inadequate trusted resources, and the need for awareness of and access to mental health resources for family members and communities most impacted by firearm injury. Future studies should evaluate whether community capacity building, digital health delivery, and trauma-informed public health campaigns could overcome these barriers to mitigate the emotional trauma of firearm injuries to reduce health disparities and prevent future firearm violence.

## Key Points

**Question:**

What are the facilitators and barriers to survivors of firearm injury seeking mental health care after injury?

**Findings:**

In this qualitative study of 18 survivors of firearm injury, survivors expressed a preference to obtain emotional support from their family members instead of mental health practitioners. Barriers included distrust in practitioners, practitioner lack of cultural congruence, and stigma in seeking mental health care.

**Meaning:**

Findings illustrate the consequences of stigma and fear when seeking mental health services, inadequate trusted resources, and the need for awareness of and access to mental health resources for family members.

## Teaser

This qualitative study evaluates the facilitators and barriers to survivors of firearm injury seeking mental health care after injury.

## Introduction

Firearm injury is associated with increased severity of adverse health outcomes^1,2^ and mental health needs,^3,4^ further compounding existing health and racial inequities among survivors and communities.^5,6,7,8^ Adult and pediatric survivors experience high rates of posttraumatic stress disorder, anxiety, and depression.^3,4,9,10^ Several survivors have expressed the need for accessible mental health services, both for themselves and their families.^11,12,13,14^ Effective treatments, such as psychotherapy and medications, exist for trauma-related mental health disorders; however, a gap remains in our understanding of how best to connect firearm injury survivors to mental health services.

Limited research has examined mental health utilization following a firearm injury using health care records.^4,9,15,16,17^ Song and colleagues^18^ estimated a 51% increase in psychiatric disorders 1 year after injury compared with controls, suggesting increased mental health needs for this population. Other studies, however, reported approximately 60% to 70% of survivors do not receive mental health treatment per health records.^4,19^ These observations raise questions about why some survivors engage in mental health care services while others do not and how to ensure access to those who would benefit.

Interventions that leverage the time in the hospital following a firearm injury to connect survivors with needed clinical, social, and mental health services, such as hospital-based violence intervention programs (HVIPs) have demonstrated success in reducing violent reinjury rates.^20,21^ Violence intervention specialists typically have personal experience with violence and strive to establish rapport with survivors.^22^ These programs, however, are not implemented in all (or even most) trauma hospitals, are limited in mental health resources, and often experience low engagement due to systemic barriers such as distrust in hospital staff, hospital workflow, and difficulty connecting with survivors after hospital discharge.^21,23,24^

Several qualitative studies of firearm injury survivors have described significant barriers when seeking clinical care after injury, including a general lack of trust in practitioners, inadequate patient-clinician communication about needs after discharge, lack of transportation to medical services, and difficulty identifying and navigating mental health services.^11,12,13,14,25^ An important limitation of existing studies is a lack of focus on mental health care utilization and participant engagement in support services. These studies may not reflect the barriers encountered by survivors not already connected with such services.

To address these research gaps, we interviewed firearm injury survivors from a community-based sample to identify facilitators and barriers to seeking mental health care. Secondary aims included exploration of their lived experiences after injury related to the emotional consequences of their shooting. This study will inform current programs on ways to improve connection to care after injury and how to promote community healing from firearm violence.

## Methods

### Study Design

This is a qualitative research study of a community-based sample of firearm injury survivors. Data were collected through semistructured interviews and inductively analyzed for common themes.^26^ The study was conducted in 2021 and 2022 in Indianapolis, Indiana. Institutional review board approval was obtained through Indiana University. The study followed the Standards for Reporting Qualitative Research (SRQR) reporting guidelines.^27^

### Selection of Participants

Participants were recruited via community partners, word of mouth, and snowball sampling. Three participants who were approached declined to participate in the interview. After each interview, the participants were asked if they knew anyone who would like to participate in the study. Eligibility criteria included surviving an intentional firearm injury determined by the participant, being shot within Indianapolis, being aged 13 years or older, and speaking English. After the first 10 interviews, we began purposeful sampling according to age and sex to recruit individuals who were younger and female to obtain different perspectives. Participants were informed the interview would be digitally recorded and assured that no identifying information would be collected. Participants were enrolled following oral informed consent procedures and were paid a $50 Visa gift card for their time.

### Interviews

Trained interviewers conducted 18 semistructured interviews. The interviewers were 2 community-based, Black, male and female violence prevention specialists (A.B.) (14 interviews), and the principal investigator (L.M.) (4 interviews). The interviews lasted 27 minutes to 105 minutes and were conducted within private spaces in the community, via Zoom, or within a private room at the community-based organization headquarters. The interview instrument was adapted from prior studies with a community-based organization to ensure the questions were relevant to the Indianapolis community.^13,28^ Participants were asked about their history with firearm violence, their life and experience after hospital discharge, their sources of past and current emotional support, their clinical and mental health care utilization, and their desired engagement with mental health care after injury. Descriptive data on race, sex, and age at time of shooting were self-reported by participants through a pre-interview survey.^28^ Race was included because of known disparities in firearm injuries.

### Data Analysis

Each recorded interview was transcribed verbatim and imported into NVivo software version 12 (Lumivero).^29^ Upon transcription, interviews were inductively analyzed by 3 independent coders (L.M., D.O., and a research assistant).^26^ Transcripts were read multiple times to identify common themes and subthemes. Each code and theme were discussed by 2 authors, and disagreements were deliberated until discrepancies were resolved. Descriptions of reoccurring themes were aggregated and represented with direct quotes from participants to provide deeper context for each theme.^30^ Data were analyzed from August 2022 to June 2023.

## Results

### Qualitative Themes

We interviewed 18 participants; 16 were male (89.0%), and 14 (77%) were aged between 13 and 24 years. The majority of participants (11 participants [61%]) were interviewed within 5 years of their shooting. All participants (Table 1) experienced physical consequences and emotional trauma from their shooting and lived with a milieu of chronic stress. Four main themes and 9 subthemes emerged from the data regarding barriers and facilitators to mental health utilization, emotional healing, and the emotional consequences of firearm injury on participants (Table 2).

**Table 1.** Participant Demographics, Survivors of Firearm Injury in Indianapolis, Indiana, 2021 to 2022

Variable	Participants, No. (%) (N = 18)
Sex	
Male	16 (89.0)
Female	2 (11.1)
Age group, time of shooting,y	
13-17	7 (38.9)
18-24	7 (38.9)
25-29	2 (11.1)
30-34	2 (11.1)
35-39	0
40-44	0
45-49	0
≥50	0
Age group, time of interview,y	
13-17	1 (5.56)
18-24	5 (27.8)
25-29	2 (11.1)
30-34	2 (11.1)
35-39	2 (11.1)
40-44	0
45-49	1 (5.56)
≥50	5 (27.8)

**Table 2.** Main Themes and Subthemes of Barriers and Facilitators to Mental Health Care Among Survivors of Firearm Injury in Indianapolis, Indiana

Main themes and subthemes	Contributing participants, No. (%) (N = 18)	Sample quote(s)
Do not need help		
Do not need mental health services	9 (50)	“I didn’t feel like it would help me. And the real fact is I didn’t feel like it would make me walk again, so what’s the point of doing that? It wouldn’t help me like that, that’s really what I was telling myself. That’s what... Yeah.” (Youth male 13)
Unwillingness to talk		
Lack of trust	10 (56)	“Again, this is about trusting people, and it’s... People like to run off and tell stuff, and even if they are professionals, it’s not their job, whatever, I still don’t like talking to people, and that’s just about me, I just don’t like talking.” (Youth male 8)
Fear/code of the streets	7 (39)	“I think I need counselling too. So I can sit down and talk about shit. Cause I’ve got a lot of shit. But then at the same time there’s so much shit that I could talk about or know about. If I talk about it I might get locked up, or somebody else might get locked up. You know what I’m saying, so?” (Youth male 4)
Family members and informal healing		
Family members/informal healing	15 (83)	“They just talked to me. They talk to me. I talk to them too, my friends, but they’re not like no normal street person. We all cool. Like we talk about our feelings. They’re gonna listen to you. If you’re expressing your feelings to them, they’re not going to laugh at you. They going to listen.” (Youth male 3) “Well, my mom thought I was [suffering emotionally]... She sent me to mental health classes sometimes. She sent me to a mental health clinic out of town.” (Youth male 5) “Yes. But I didn’t get connected through nobody but a friend of a friend. You know what I’m saying? They connected me to them, I spoke to them, told them my story and what I’ve been through, and whenever I needed to talk, they were there.” (Adult female 6)
Family impacted	17 (94)	“Man, I think it messed my mama up more than anything. She’s still so paranoid, I can’t help that. You know, I’m her only child, for one. I’m her only, only child. My dad, I don’t know if you know, but my dad, all of his brothers... All of his brothers on his dad’s side, you know, the majority of them died before they were 30. It’s only a handful of them that made it past. Well, it ain’t even a handful, it’s only a couple of them that made it past 30. And I wanna say, what, 3 of them died out of, what, 6 or something like that. Three of ‘em... Yeah, so I think like 3 of them died before 30. And it was from some kind of monetary intent. It had her paranoid, it had him paranoid. I was 29. So knowing that his brothers were dying before 30 and his son is 29, it was like, ‘Oh man.’ To him, it was like a curse. And that I’ve never seen that much amount of support from my pops, you feel me, but I could tell that it tapped something in him, like that’s all that was on his mind.” (Adult male 1) “I need somebody to come outside and help me, pick me, like, jump me up the curve, ‘cause I didn’t get the [wheelchair] accommodation, so they have to come out and help me. And so that’s one way they was impacted, and now I live, I sleep downstairs in the living room….My brother been helping me taking my wheelchair apart before school, every morning doing that, it kind of slows us down in the morning, he’d be late for his class, so that’s one way. He just helps sometimes, but I don’t always need help, but they do play a big part to help me.” (Youth male 13) “Well, like I said, it definitely, probably affected my son a lot. But I was thinking it affected my marriage a lot. I think she probably became unsafe a little bit on, ‘Damn, what the hell had you been doing shooting?’ So, and then, I think it affected my dad a lot. But my dad, he was battling with cancer at the time. So, it was kinda like, he was in the hospital right before, and then the next thing you hear about me is getting shot. So, it just really, I think it kinda like... Yeah, brought something on him.” (Adult male 9)
Credible messengers		
Formal mental health services	7 (39)	“Well, I’ve been attending it [therapy], I’ve been in it before the incident happened. So I was dealing with Dr. Smith office, and now I’m through mental health clinic, I deal with mental health clinic right now. And it was before this even happened, I was dealing with it anyway.” (Adult male 10)
Unhelpful	11 (61)	“She’ll sit there and talk and listen to me about stuff. It’s just, I feel the only thing about her is that we’re from 2 different worlds.” (Adult male 1) “She like to LOL a lot, like what’s laugh out loud? What you laughing for? She talking about, ‘Oh, that’s just how I talk.’ Well find somebody else. And then I feel like she don’t even really care, bro. She always just wanna meet. I feel like she meet with me just so she could turn it in to her supervisor and get paid. I feel like it’s only about a check with her, it’s not genuine. She don’t... I feel like she don’t care about.” (Adult male 14)
Someone to listen/understands life	11 (61)	“Who overcame their trauma and things like that so they can relate and connect with the people ‘cause nobody is gonna listen to somebody who has never been in their shoes, never been in any type of situation. You know what I’m saying?” (Adult female 6) “Somebody’s that’s not gonna judge. ‘Cause I know a lot of people, because they got the certifications, that don’t mean you know what you’re talking about at all. So, I am very judgmental when it comes to that, because we’ve got the doctors that are actually knowing what’s going on, they know how to talk to you and stuff like that. Then you got the quacks where they sit there and they act like they know what you’re talking about, they act like they’ve been through it and they’re uppity. They don’t know nothing about street life. They’re not from areas like where I’m from. They are from [suburbs], places like that. Places where they don’t have violence. And it is mine and my sister’s neighborhood where it pops Indianapolis off, so.” (Adult male 11)
Interview process	6 (33)	“Yeah, I just, I don’t know. I feel it’s nice to tell the truth and be anonymous about it, ‘cause I haven’t had the chance to. I think I feel good about this conversation. It was nice. Honest about myself... About what was going on with my family and not talking to them about this incident, but other things. I didn’t realize I haven’t really talked about it since it happened with them. I literally have not talked about the shooting at all since 2017 with my family. And it’s like, ‘Wow. I didn’t even peep that I hadn’t talked to them about it.’ And my boyfriend, he knows about it, but I don’t really talk to him about it as well, either. I guess it’s just something I really put off and really only keep to myself nowadays. So it was good to figure out that that’s something I need to work on ‘cause it does still affect me today.” (Youth female 12)

The first main theme was that participants felt they did not need help**.** Despite their self-described emotional symptoms, half of the participants stated they did not have a mental health problem and noted an expectation to quickly move on from their shooting. Adult male 8 stated, “Really, I was still doing the same thing, running around these streets. So, it [the shooting] didn’t affect me none.”

The second main theme was their unwillingness to talk. Participants expressed they were unwilling to discuss their shooting with a therapist due to a lack of trust among people outside their immediate network. Youth male 7 noted, “I didn’t really rely on the doctor, I relied on my mom a lot. It’s just a thing about me with other people. I don’t trust a lot of people, and with that situation, it just really boosted up my anxiety a lot, and upped the ante for me not to trust people more.” Participants were fearful of getting in trouble with the police and even viewed speaking to a practitioner as snitching or informing on someone else. Youth male 5 said, “It because back then when you’re in the street you any time might, keeping the code of the street you ain’t death no more than you ain’t trying to get no help. You ain’t trying to ask nobody for no help or nothing, or tell the police. That’s called snitching.” When the interviewer asked, “And so going to get services leads to…,” the participant replied, “Snitching.” Therefore, most participants chose to not seek care or talk to anyone outside their trusted network about their injury.

The third theme related to family members and informal healing. Nearly all participants discussed how they received their own physical and emotional healing through family members and friends. Adult female 6 stated, “Well, my mom, I’ll call her ‘cause I don’t really go around her. So I’ll just call her and tell her what’s on my mind. She’ll give me a positive response. My man, I talk to him, pull him to the side and tell him what’s going on. He’ll give me a real-life response, and my sister, she gives me a spiritual response.” Family members and informal networks also facilitated connections to formal mental health services through word of mouth and family encouragement. Adult male 15 said, “Yeah. I met with my sister’s doctor. Yeah, yeah. My family doctor, my family. That was… It got me on my feet.” Participants also discussed how their shooting impacted their families emotionally during the period after injury. Adult male 4 stated, “Yeah, because it impacts everybody. The more I’m impacted, it impacts... I got 3 kids, 3 baby mamas and a girlfriend. Man, all 4 of them ladies is impacted and affected by what’s affecting me. And then my kids... It trickles down. You know what I’m saying? And then my kids are affected because what’s affecting me affects them and then their mamas and then the mamas affect the kids.” Additionally, participants discussed the need to relocate residences due to fear since the offender knew where they lived, which put additional emotional and financial burdens on their families. Youth male 3 said, “Because my mom, she was scared. Me and my sisters and my girl and my mom, we all lived there. So, my mama moved because she didn’t want nobody come to our house or shoot up the house or something while her daughter’s in there. How it impacted my family ‘cause like we don’t have a home, you know we homeless. My mum over there, I’m over here, we mean... Everything got messed up. Everything just got torn apart split up… She didn’t wanna live where she ain’t feel safe.”

The fourth theme related to credible messengers and trusted space. Participants that attended formal mental health services following their injury were connected through a credible messenger, such as a prior mental health or primary care practitioner or a violence intervention specialist through the local HVIP. Participants expressed they had an established trusted connection with the practitioner, felt they were being heard, and that the practitioner understood their needs. Youth male 2 said, “I was connected with him before I got shot, so he was my doctor before I even got shot, but by me getting... That’s why I think he did what he did because we had rapport already, and he knew I wasn’t just BS’ing him and I think... This is just me thinking, but I think we’re humans, we made connection.” On the contrary, mental health practitioners were perceived as unable to be helpful to survivors if they were not viewed as trustworthy and relatable. Adult male 1 stated, “I told them in the group this, any my counselors and everything, they had no answer. What do I do when I’m out with my family? Because like I said, I wanna go on my trips, I wanna go to the pool, I wanna go out to eat, I wanna go to baseball. And I’ll walk, and it’s one thing if I see him [offender] from a distance. It’ll be like, ‘Oh, ya’ll we gotta go.’ It’s another thing when I’m up and I look up, and he look up [offender] and see me. Then its like, ‘damn.’”

When asked about their ideal mental health services, participants discussed the desire for 1-on-1 therapy, group therapy, telehealth services, and that services should be provided in the heart of the Black community, while others wanted to seek services outside the city (ie, suburbs). No matter the mechanism, participants expressed the desire for someone who understood them, who understood their life and community, who was not going to judge, and, ultimately, someone who resembled them. Youth male 2 said, “It would have to be somebody like me. Someone they will really interact with. Okay, Boom. Now I got your attention versus this guy that’s got this button-up on, man. I got your attention.”

Lastly, some participants conveyed the interview process provided an opportunity for them to share their story within a confidential space and gave them a level of emotional relief they had not experienced before. Adult male 1 said, “Yeah, this is support. Just being able to go somewhere and talk is support. Even if y’all doing research, for me it’s therapy.”

## Discussion

This qualitative analysis provided novel perspectives on firearm injury survivors’ experiences with healing from the emotional consequences of their shooting. In addition to key facilitators and barriers to seeking formal mental health care, major findings included the importance of informal sources of emotional support for firearm injury survivors from family, friends, and informal networks. Second, the results highlighted the emotional consequences the shooting had on survivors’ families—particularly mothers, partners, and children—by inflicting emotional distress, anger, and fear for their safety. This led several families to move residences and to live with other family members. These findings highlight the ripple effects of firearm injury beyond the direct survivor to family members, adding to the growing literature in this area.^3,11,18^

This study identified 3 main themes and barriers to survivors accessing mental health services: perceived lack of benefit of mental health services, distrust in practitioners, and fear of stigma and punitive backlash. Most importantly, firearm injury survivors overwhelmingly expressed their desire to navigate firearm injury recovery on their own, and half believed they were adequately coping without formal mental health services. Survivors doubted the ability of practitioners to give helpful advice, as practitioners did not understand their life and community environments. Survivors expressed a general lack of trust during the period after injury, which was intensified by a history of complex trauma.^31^ This distrust extended to service systems meant to support survivors, including mental health practitioners.

In addition to distrust, survivors in this study expressed a fear of getting in trouble or being viewed as a snitch within their communities for talking with a mental health practitioner. The antisnitching code of silence is an informal rule within the subcultural code of the streets*.* People who break the code may be susceptible to physical injury themselves.^32^ The antisnitching code has been largely applied to speaking with the police and victim’s services offered within police departments.^32,33,34,35^ Survivors often perceive blurred lines between medical professionals and police at the time of their firearm injury.^36,37^ Our findings demonstrate the antisnitching code also extends to mental health practitioners and is a crucial barrier to survivors of firearm injury seeking potentially helpful mental health services.

Our findings also demonstrated 2 key facilitators to seeking mental health care: a safe space/environment for the survivor to express themselves and a credible messenger. One-third of survivors expressed that the research interview was the first time they had discussed their shooting, and they felt comfortable doing so because of the privacy and anonymity. Survivors also expressed the importance of cultural congruence and having a credible messenger among their care team. This has been noted as a criteria for violence intervention specialists and is supported in other studies on firearm injury survivors.^13,25^ Similarly, our findings extended this concept to include clinicians with whom survivors had a prior connection; often a pediatrician or other primary care practitioner. Such practitioners served as key facilitators to mental health care after injury among survivors in this study. Participants described high levels of trust in these practitioners, attributed to their general understanding of the survivors’ life and lack of judgement. Participants valued the ability to express their stories freely with them. These findings suggest the importance of engagement with primary care practitioners and regular health care screening for risk of firearm injury.^38^

Our findings have several implications to improve the connection to care and for future research. Given survivors’ preference for informal services, we need to explore new paradigms for delivering mental health services, such as digital health, community-based and family-led support. Digital nonemergency transportation services (eg, Uber Health) for survivors and families have demonstrated success in better connecting patients with HVIPs.^25^ Other mobile-based applications have shown utility in implementing mental health-based interventions among patients with trauma.^39^ For instance, a technology-based, stepped, access-to-care model has shown high rates of acceptability and engagement among trauma survivors, including those impacted by violence.^39^ Encouragingly, similarly high rates of engagement in recommended treatment services have been observed among participants regardless of racial or ethnic background.^40^ Adapting such interventions for survivors of firearm injury and families is a clear future direction.

Family members were also identified as a main source of support after injury. Family involvement is often a key factor in preventing mental health concerns and promoting behavioral health recovery following trauma.^41^ In some cases, existing support from families and peers may be sufficient in helping survivors weather the aftermath of a trauma. In other cases, formal treatment services are warranted. Future work should consider ways to raise awareness among family members regarding mental health resources and how best to connect their loved ones with effective services. Additionally, programs should expand mental health resources to family members.^3,11^

These findings also highlighted the importance of tailoring interventions to an individual’s needs and cultural values,^42,43^ such as incorporating culturally specific coping strategies into trauma-focused treatment, which yield better outcomes.^44,45^ Steps to achieve this include training more mental health professionals from underrepresented communities, strengthening community partnerships, and public health messaging campaigns to improve connection to care. Health care practitioners, community organizations, and faith communities can work together to increase community engagement with mental health services.^46,47,48,49,50,51,52^ Future studies should evaluate whether trauma-informed public health campaigns and community capacity building could overcome the barriers to mental health service utilization.

### Limitations

There were several limitations in this study. Recruitment among this population was challenging due to mistrust and fear of getting in trouble; we mitigated this through our partnership with our community-based organization; conversely, our sample size is smaller. We met saturation after 15 interviews; however, we were only able to recruit 2 female participants and were unable to reach saturation among female survivors. We noted some differences among female survivors, and examining experiences among female survivors is a clear direction for future research. The majority (61%) of participants were interviewed within 5 years of their shooting; other participants’ memories of events may have changed given the amount of time between their shooting and the interview.

## Conclusion

This qualitative study illustrated the fear of stigma, inadequate trusted resources within communities, and the need for awareness of and access to resources for families and communities impacted by firearm violence. It is vital to improve engagement with mental health care with credible messengers through multiple delivery mechanisms, such as digital health, community-based interventions, and family support. Healing the emotional trauma associated with firearm injury for both survivors and families can reduce health disparities, prevent future firearm violence, and improve population health.

## Notes

Supplement.
Data Sharing Statement
