# Supplementary material for: Engagement With Mental Health Services Among Survivors of Firearm Injury
Source: JAMA Netw Open. 2023 Oct 30;6(10):e2340246. doi: 10.1001/jamanetworkopen.2023.40246 (PMC10616725; doi:10.1001/jamanetworkopen.2023.40246)
Supplement: Supplement. — Data Sharing Statement [file jamanetwopen-e2340246-s001.pdf]

Data Sharing Statement

Magee. Engagement with Mental Health Services Among Survivors of Firearm Injury. *JAMA Netw Open*. Published October 30, 2023. doi:10.1001/jamanetworkopen.2023.40246

Data
Data available: No

Additional Information
Explanation for why data not available: Data were collected from participants with the understanding their narratives in full would not be shared outside the research team, therefore we choose not to share the data publicly to ensure the confidentiality of our participants. Data are not available to share.
